# Antibacterial Size Effect of ZnO Nanoparticles and Their Role as Additives in Emulsion Waterborne Paint

**DOI:** 10.3390/jfb15070195

**Published:** 2024-07-17

**Authors:** Imroi El-Habib, Hassan Maatouk, Alex Lemarchand, Sarah Dine, Anne Roynette, Christine Mielcarek, Mamadou Traoré, Rabah Azouani

**Affiliations:** 1 Laboratoire des Sciences des Procédés et des Matériaux (LSPM-CNRS UPR 3407), Institut Galilée, Université Sorbonne Paris Nord, 99 Avenue Jean-Baptiste Clément, 93430 Villetaneuse, France; e.imroi@hubebi.com (I.E.-H.); alex.lemarchand@lspm.cnrs.fr (A.L.); sarah.dine@lspm.cnrs.fr (S.D.); mamadou.traore@lspm.cnrs.fr (M.T.); 2 Ecole de Biologie Industrielle (EBI), EBInnov^®^, 49 Avenue des Genottes-CS 90009, 95895 Cergy Cedex, France; h.maatouk@hubebi.com (H.M.); a.roynette@hubebi.com (A.R.); c.mielcarek@hubebi.com (C.M.)

**Keywords:** ZnO nanoparticle, antibacterial activity, nanoparticle size effect, bacterial growth, antibacterial paint

## Abstract

Nosocomial infections, a prevalent issue in intensive care units due to antibiotic overuse, could potentially be addressed by metal oxide nanoparticles (NPs). However, there is still no comprehensive understanding of the impact of NPs’ size on their antibacterial efficacy. Therefore, this study provides a novel investigation into the impact of ZnO NPs’ size on bacterial growth kinetics. NPs were synthesized using a sol–gel process with monoethanolamine (MEA) and water. X-ray diffraction (XRD), transmission electron microscopy (TEM), and Raman spectroscopy confirmed their crystallization and size variations. ZnO NPs of 22, 35, and 66 nm were tested against the most common nosocomial bacteria: *Escherichia coli*, *Pseudomonas aeruginosa* (Gram-negative), and *Staphylococcus aureus* (Gram-positive). Evaluation of minimum inhibitory and bactericidal concentrations (MIC and MBC) revealed superior antibacterial activity in small NPs. Bacterial growth kinetics were monitored using optical absorbance, showing a reduced specific growth rate, a prolonged latency period, and an increased inhibition percentage with small NPs, indicating a slowdown in bacterial growth. *Pseudomonas aeruginosa* showed the lowest sensitivity to ZnO NPs, attributed to its resistance to environmental stress. Moreover, the antibacterial efficacy of paint containing 1 wt% of 22 nm ZnO NPs was evaluated, and showed activity against *E. coli* and *S. aureus*.

## 1. Introduction

Nosocomial infections (Nis) represent common complications among patients admitted to intensive care units (ICUs), with an incidence ranging from 5% to 10% in Europe and America [1]. These infections result from antibiotic overuse, leading to microbial resistance. Antimicrobial resistance (ARM) occurs when microorganisms, including bacteria, viruses, fungi, and parasites, become able to adapt and grow in the presence of medications that once impacted them [2]. For example, the 2022 Global Antimicrobial Resistance and Use Surveillance System (GLASS) reported that in 2020, one in five cases of urinary tract infections caused by *E. coli* showed reduced susceptibility to standard antibiotics such as ampicillin, co-trimoxazole, and fluoroquinolones [3]. Antibiotic resistance increases the risk of incurable infections. The mechanisms of antimicrobial resistance include decreased drug inactivation or modification through enzyme production that destroys or alters antibiotics, alters target or binding sites such as penicillin-binding or ribosomal-binding proteins, and alters metabolic pathways, like the ability of enterococci to absorb folic acid from the environment, allowing them to bypass the effects of trimethoprim-sulfamethoxazole [4].

According to the OECD, the rate of antibiotic resistance is set to double by 2035 compared with 2005 [5]. Antibiotic-compromised efficacy leads to the exploration of alternative approaches, such as using metal oxide nanoparticles, notably ZnO nanoparticles, to combat antimicrobial resistance and prevent the emergence of resistant pathogens.

Metal oxide nanoparticles (NPs) have emerged as promising candidates for addressing ARM due to their unique physicochemical properties and high surface-to-volume ratio, which enable them to exert diverse modes of action (MOAs) against microorganisms. The mechanism of action of NPs encompasses chemical, physical, and combined interactions with bacterial cells. The chemical mechanism involves a series of steps, beginning with the release of ions and the subsequent generation of reactive oxygen species by NPs [6,7,8]. Metal ions are adsorbed on the cell membrane and later interact with the functional groups (-COOH, -NH_2_, and -SH) of nucleic acids to deactivate the enzyme, causing a change in cell structure [9]. Elevated ROS have many effects on bacteria, and lipid peroxidation is one of them [8]. The physical interaction involves direct interactions between NPs and microorganisms. NPs can physically interact with bacterial cell walls, causing structural damage that leads to cell lysis. The combined mechanism involves electrostatic interactions between NPs and the cell membrane. This electrostatic interaction can cause the depolarization of the bacterial cell membrane, leading to potential membrane loss and cell disruption. In addition, NPs can also be internalized via endocytosis, nonspecific uptake, membrane diffusion, and adsorption [10,11]. Consequently, metal oxide nanoparticles show promise as potent antimicrobial agents [9,12] due to their ability to use multiple mechanisms of action to combat bacterial resistance.

Zinc oxide is attracting attention as a compelling metal oxide material due to its biocompatibility, easy synthesis, chemical stability, high abundance, and affordability [13,14,15]. These characteristics make it an ideal candidate for various biomedical applications, including its potential use in combatting bacterial infections. Previous studies have demonstrated the effectiveness of ZnO nanoparticles against a broad spectrum of pathogenic microorganisms, including Gram-positive and Gram-negative bacteria [16,17,18]. Their mode of action relies, in part, on the production of reactive oxygen species [19,20,21,22,23], which initiate the oxidation of cellular components, the disruption of bacterial membranes [11,23,24,25,26,27], direct contact [28,29,30,31], and internalization [16,22], ultimately resulting in cell death.

Enhancing the antibacterial potential of ZnO NPs is crucial for effectively preventing infections. The existing literature provides insights into how ZnO nanoparticle characteristics, such as shape [22,32,33,34,35,36,37,38], size [21,39,40,41,42], concentration [43,44], and surface modification [43,44,45,46], impact their antimicrobial activity. The effects are studied using techniques like disk and well diffusion assays, optical density measurements at short timescales (≤6 h), and the determination of minimum inhibitory concentration (MIC) and minimum bactericidal concentration (MBC). However, our study focuses on the effect of nanoparticle size on bacterial growth kinetics. Smaller sizes mean a high surface area for the materials. This increase in specific surface area makes them more reactive, resulting in a high concentration of surface particles capable of interacting with electrons and holes. This unique characteristic offers more sites where electrical charges can accumulate, resulting in an increased surface electric charge, which leads to strong electrostatic interactions between ZnO NPs and bacterial membranes. The particle’s charge can influence its biocompatibility and ability to traverse biological barriers [46]. The small dimension of these materials also makes them more abrasive [47]. Additionally, the sizes of NPs (ranging from 1 to 100 nm) are comparable to the size of protein globules (2 to 10 nm), the diameter of the DNA helix (2 nm), and the thickness of cell membranes (10 nm) [10]. Thus, the decrease in their size allows them to enter cells and cell organelles.

Previous studies have demonstrated that reducing nanoparticle size enhances their antibacterial activity [20,38,48]. Babayevska et al. [20] found that ZnO nanoparticles were more effective than microparticles against *Escherichia coli* and *Staphylococcus aureus*. They explained this difference by examining the mechanism through ROS measurements, and found that NPs produced more ROS than microparticles. Raghupathi et al. [49] found a superior antibacterial activity of ZnO NPs at reduced sizes. They compared the growth curves of bacteria in the presence of ZnO NPs of various sizes (30 nm, 88 nm, 142 nm, and 212 nm) at a concentration of 6 mM during 6-h cultures. Despite these efforts, little research has been carried out to compare the bacterial growth kinetic across different sizes to understand the size-dependent mode of action.

The originality of this work lies in studying the influence of NPs’ size on bacterial growth kinetics, focusing on the latency period and specific growth rate of bacteria. Determining these parameters sheds more light on the effect of NPs on the growth mechanisms of bacterial strains. Additionally, this study evaluates the antibacterial activity of paint formulated with synthesized NPs. The bacteria kinetic study was conducted on Gram-negative and Gram-positive bacteria by monitoring changes in bacterial optical density over a 24-h culture period with different NP sizes and concentrations. The specific growth rate and the latency period of bacteria were calculated using optical density data. Before this, the impact of the size of synthesized ZnO NPs on their antibacterial activity was preliminarily examined by determining their minimal inhibitory concentration (MIC) and bactericidal concentration (MBC). Finally, the conservation of the antibacterial activity of synthesized ZnO NPs in a paint formulation as a biocidal agent was evaluated.

## 2. Material and Methods

### 2.1. Materials

Zinc acetate dihydrate (≥99%), butan-1-ol (≥99.5%), acetone (≥99.5%), and silicone oil were obtained from Sigma-Aldrich, Darmstadt, Germany. Isopropanol (≥99.5%) was acquired from Acros Organics (Geel, Belgique). Monoethanolamine (100%) was obtained from Emprove (Darmstadt, Germany). Sodium polyacrylate (PAAS) was supplied by *Cosmedia* sp. (Ludwigshafen, Germany). Muller–Hinton broth and Trypto Soybean Casein were purchased from DIFCOTM (Sparks, MD, USA) and BioMerieux (Darmstadt, Germany). Powder paint was supplied by Dolci (Auvergne-Rhône-Alpes, France) and Eugon LT SUP by BioMerieux (Auvergne-Rhône-Alpes, France).

### 2.2. Synthesis of ZnO Nanoparticles

The protocol applied to obtain ZnO nanopowders of various sizes has been described in detail in a previous article [50]. A zinc precursor, with a concentration of 0.1 M, was dissolved in 100 mL butan-1-ol in the presence of a complexing agent, monoethanolamine (MEA). Water was introduced into the reaction medium to induce the precipitation of the NPs and to obtain nanopowders with a satisfying yield. NP size was mainly controlled by the ratio of zinc ions to complexing agent [Zn^2+^]/[MEA] and the hydrolysis rate [Zn^2+^]/[H_2_O]. The nanopowders were collected via centrifugation, followed by three washes with isopropanol and acetone to remove organic residues. Next, 2 wt% nanopowders were dispersed in Mueller–Hinton (MH) broth, followed by ultrasonic treatment in a J.P. Selecta ultrasonic bath at 150 W and 42 kHz for 1h in the presence of 0.4 wt% PAAS used as a dispersant. The protocol for preparing stable suspensions of NPs was inspired by that of Luo et al. [51].

The synthesis parameters used to prepare ZnO nanopowders are summarized in Table 1.

### 2.3. ZnO Nanoparticles’ Characterization

The phase identification and microstructural characterization of ZnO nanopowders were carried out using X-ray diffraction (XRD) measurements on an EQUINOX 100 XRG 3000 diffractometer manufactured by INEL (Orléans, France)using a monochromatic cobalt source (λ Kα1 (Co) = 1.788976 Å). Diffraction patterns were processed using the Rietveld method implemented in MAUD (Material Analysis Using Diffraction) software (V2.998). Peaks were fitted according to a standardized procedure. The parameters refined were cell parameters, NP size, and the presence of microstrain. The crystallographic information file for ZnO wurtzite was obtained from the Crystallography Open Database (Ref 2300450). In the final step, an arbitrary texture option was used to optimize the quality of the Rietveld pattern refinement. This characterization was completed using Transmission Electronic Microscopy (TEM) analysis performed on a JEOL 2011(Tokyo, Japan) equipped with a Gatan Imaging Filter (DIF) 200 (Pleasanton, CA, USA). Raman spectroscopy measurements were also carried out on the powder samples using an HORIBA Jobin-Yvon HR800 spectrometer (Palaiseau, France) with an excitation wavelength (λ = 633 nm) to confirm the formation of ZnO NPs.

### 2.4. Minimal Inhibitory Concentration (MIC)

The MIC is the lowest concentration of NPs that prevents visible bacterial growth during incubation. Different concentrations of NPs were tested against bacteria to determine the MIC. These concentrations were prepared using microdilutions of the ZnO NP stock suspension. In total, 200 μL of each sample were then inoculated on a 96-well microplate in the presence of a final microorganism concentration of 4.67 Log_10_ CFU/mL. Physiological water was added to the empty microplate wells to ensure good humidity. Samples were incubated in a FLUOstar Omega spectrophotometer for 24 h with double orbital shaking at 300 rpm to monitor changes in optical density. Experiments were performed in triplicate. Negative controls included culture medium, NP solution, and PAAS solution. Positive controls consisted of PAAS solution in contact with microorganisms without NPs, as well as microorganisms in MH medium alone, without dispersant or NPs. The MIC was evaluated as the lowest concentration at which the optical density of bacteria remained constant.

The Biological Resource Center of Institut Pasteur (CRBIP) in Paris, France, provided the bacterial strains, which included *Escherichia coli* ATCC 8739 (CIP 53 126), *Pseudomonas aeruginosa* ATCC 9027 (CIP 82118), and *Staphylococcus aureus* ATCC 6538 (CIP 53 156). The bacteria were previously cultivated on MH agar from cryotubes stored at −80 °C. Incubation temperatures were set at 37 °C for *E. coli* and *S. aureus*, and at 30 °C for *P. aeruginosa*.

### 2.5. Minimal Bactericidal Concentration (MBC)

MBC is the lowest concentration of NPs resulting in at least a 99.9% reduction in cell viability. Various concentrations of NPs were incubated with bacteria in the FLUOstar at the appropriate temperature for 24h. After incubation, samples were diluted, and a volume of 1 μL was inoculated onto MH agar. The MH agar plates were then incubated at appropriate temperatures for colony counting. Percentage cell reduction was calculated according to the following formula:

(1) $$Percentage\;of\;reduction=(1-(C_{f}/C_{i}))\times 100\;in\;\%$$

(2) $$C_{i,f}=\log\left(n/\left(V*d\right)\right)$$

where

C_i_ is the initial concentration of microorganisms in contact with NPs before incubation,

C_f_ is the final concentration of microorganisms in contact with NPs after 24 h of incubation,

n is the number of colonies counted,

V is the inoculated volume,

d is the dilution factor.

### 2.6. Kinetic Growth of Bacteria

The growth kinetics of bacteria in contact with NPs were monitored by measuring the samples’ optical density (OD) over time using the FLUOstar Omega spectrophotometer at a wavelength of 600 nm. OD_600_ measurement was used as a rapid and cost-effective means of monitoring the growth of bacteria throughout their culture in contact with NPs. NP concentrations at which the optical density of bacteria was not constant during MIC determination, as explained above, were used to study the impact of NP size on bacterial growth kinetics. The lag period, the specific growth rate of bacteria, and the percentage inhibition of NPs were evaluated using OD_600_ data. The formulas have been previously cited in reference [50] and are shown below:

(3) $$\mu_{x}=\left(\ln\left(X_{2}\right)-\ln\left(X_{1}\right)\right)/\left(t_{2}-t_{1}\right)\;in\;h^{-1}$$

(4) $$G=ln(2)/\mu_{x}\;in\;hour$$

(5) $$Inhibition={(OD}_{f}^{bacteria}-({OD}_{f}^{NPs+bacteria}-{OD}_{control}^{NPs-bacteria})/{OD}_{f}^{bacteria})\times 100\;in\;\%$$

where

μ_x_ is the specific growth rate;

X_2_ and X_1_ represent biomass at times t_1_ and t_2_, respectively, during the exponential growth phase in CFU/mL;

t_2_ − t_1_ is the time corresponding to biomass growth from X_1_ to X_2_ in hours;

G is the generation time in hours;

${OD}_{f}^{bacteria}$ is the final optical density of the positive control with bacteria alone;

${OD}_{f}^{NPs+bacteria}$ is the final optical density of bacteria in contact with NPs;

${OD}_{control}^{NPs-bacteria}$ is the optical density of NPs alone without bacteria.

The conversion of OD_600_ to CFU/mL was performed using a calibration equation OD = f(X) established within the laboratory for each bacteria under the operational conditions, where X represents the biomass in CFU/mL.

### 2.7. Paint Formulation with ZnO Nanoparticles

To obtain a suitable dispersion, a preservative-free Dolci-brand paint powder was dispersed in sterile distilled water at a mass ratio of 1:1 using a EUROSTAR 20 digital stator rotor emulsifier. Next, a 1 wt% solution of 22 nm ZnO NPs was added. The paint was then applied by dip-coating it onto standard glass substrates (VWR) pre-treated with sulfuric acid to ensure better paint adhesion. The paint was then left to dry for 24 h.

### 2.8. Measurement of the Antibacterial Activity of Paints Containing Nanoparticles

The antibacterial activity of the paints was assessed following ISO 22196:2011 [52] with slight modifications. A total of 0.1 mL bacterial suspension, prepared in 1/500 NB nutrient broth (3.0 g of meat extract, 10.0 g of peptone, and 5.0 g of sodium chloride), at 6 Log CFU/mL was spread onto paint films. Samples were then covered with polypropylene film (2 × 5 cm^2^) to maintain humidity and ensure good contact between samples and bacteria. They were incubated at 35 °C under ≥90% relative humidity for 24 h. After incubation, bacterial cells were recovered from the paint films by stirring in 10 mL of Eugon LT broth in the presence of 10 g of 1 mm glass beads for 1 min. The antibacterial activity was evaluated via colony counting on agar plates. Positive controls were conducted using paint without NPs. All tests were performed in triplicate, and the antibacterial activity was calculated using the following formula [52]:

(6) $$R=U_{t}-A_{t}\;in\;{Log}_{10}\;CFU/{cm}^{2}$$

R is the antibacterial activity;

U_t_ is the average of the common logarithm of the number of viable bacteria, in cells/cm^2^, recovered from the untreated test specimens after 24 h.

## 3. Results

### 3.1. Characterization of ZnO Nanoparticles

**Figure 1:**
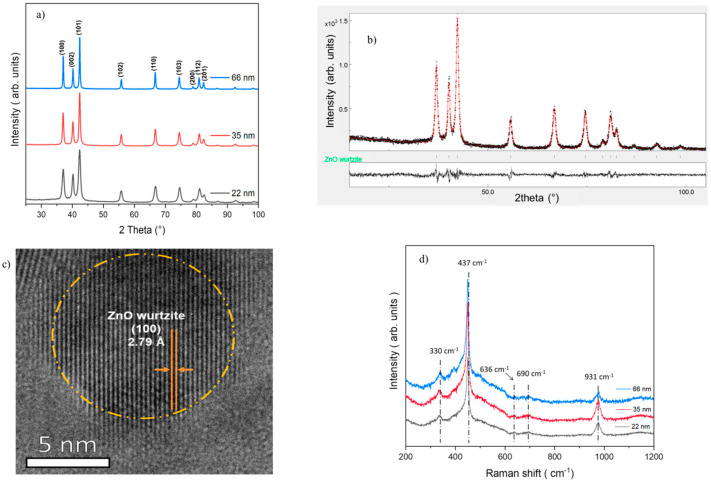
(**a**) XRD patterns of samples; (**b**) Rietveld refinement of 22 nm ZnO; (**c**) TEM of 22 nm ZnO dispersed in MH broth; and (**d**) Raman spectra of samples.

All ZnO samples are well crystallized in the wurtzite phase, as confirmed using X-ray diffraction (Figure 1a) and Rietveld refinement (Figure 1b). Rietveld refinement analysis showed the stability of the cell parameters (a, c) and revealed varying sizes—22 nm, 35 nm, and 66 nm (Table 2). In addition, the transmission electron microscopy (TEM) image of 22 nm NPs confirmed the formation of small and well-crystallized ZnO wurtzite NPs (Figure 1c). Raman spectra also confirmed the crystallinity of the samples, showing characteristic Raman modes indicative of the ZnO wurtzite structure (Figure 1d).

### 3.2. Correlation between ZnO Nanoparticle Size and Minimal Inhibitory Concentration (MIC)

**Figure 2:**
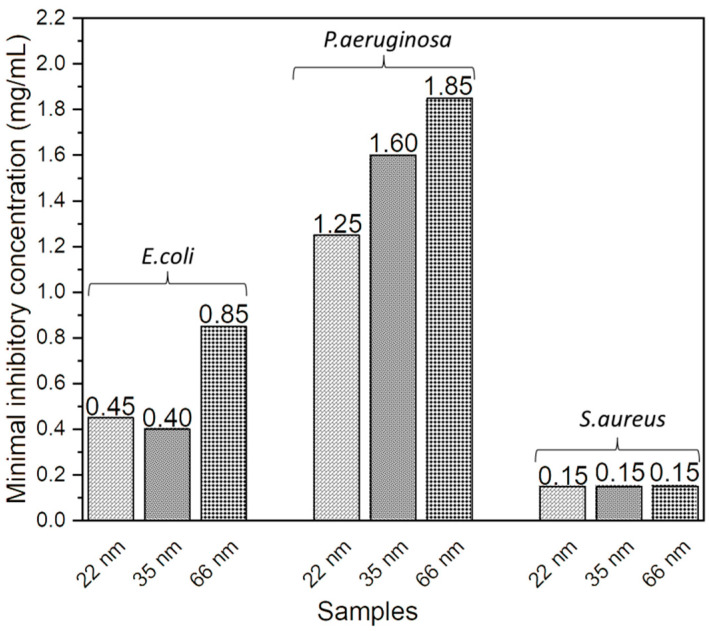
Effect of ZnO nanoparticle size on MIC against *E. coli*; *P. aeruginosa*; and *S. aureus*.

The MIC represents the lowest concentration of ZnO NPs that inhibits visible bacterial growth after the incubation period. A significant decrease in the MIC of ZnO NPs against *E. coli* was observed as their size decreased, with respective values of 0.45 mg/mL, 0.40 mg/mL, and 0.85 mg/mL for sizes 22 nm, 35 nm, and 66 nm. Similarly, a reduction in MIC was noted for *P. aeruginosa*, with values decreasing from 1.25 mg/mL and 1.60 mg/mL to 1.85 mg/mL for sizes of 22 nm, 35 nm, and 66 nm, respectively. The antibacterial activity of ZnO NPs against *E. coli* and *P. aeruginosa* increased as their size decreased. The MIC of ZnO NPs against *S. aureus* remained stable at 0.15 mg/mL regardless of their size. These results are presented in Figure 2.

### 3.3. Correlation between ZnO NP Size and Minimal Bactericidal Concentration (MBC)

**Figure 3:**
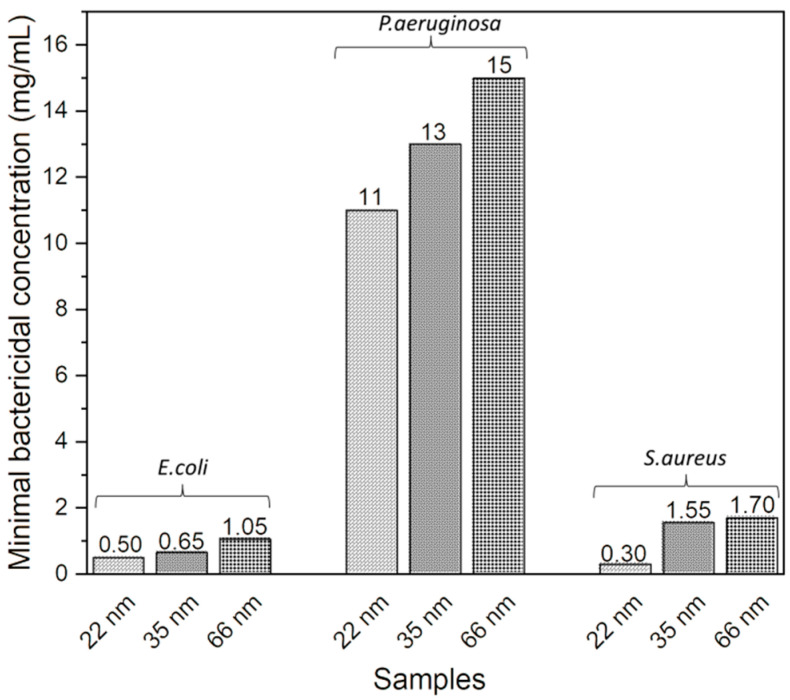
Effect of the size of ZnO NPs on their MBC against *E. coli*, *P. aeruginosa*, and *S. aureus*.

The MBC corresponds to the lowest concentration of NPs, resulting in the death of 99.9% of the initial bacterial population. It was observed that the MBC of NPs against the three bacteria decreased as their size decreased, indicating an improvement in the antibacterial activity of the NPs and confirming the results obtained previously (Figure 3). Specifically, its values were 0.5 mg/mL, 0.6 mg/mL, and 1.05 mg/mL for sizes of 22 nm, 35 nm, and 66 nm against *E. coli*, respectively. For *P. aeruginosa*, the values were 11 mg/mL, 13 mg/mL, and 15 mg/mL for sizes of 22 nm, 35 nm, and 66 nm, respectively. And for *S. aureus*, the MBC values were 0.30 mg/mL, 1.55 mg/mL, and 1.70 mg/mL for sizes of 22 nm, 35 nm, and 66 nm, respectively.

### 3.4. ZnO NP Size Effect on Bacterial Growth Kinetics

**Figure 4:**
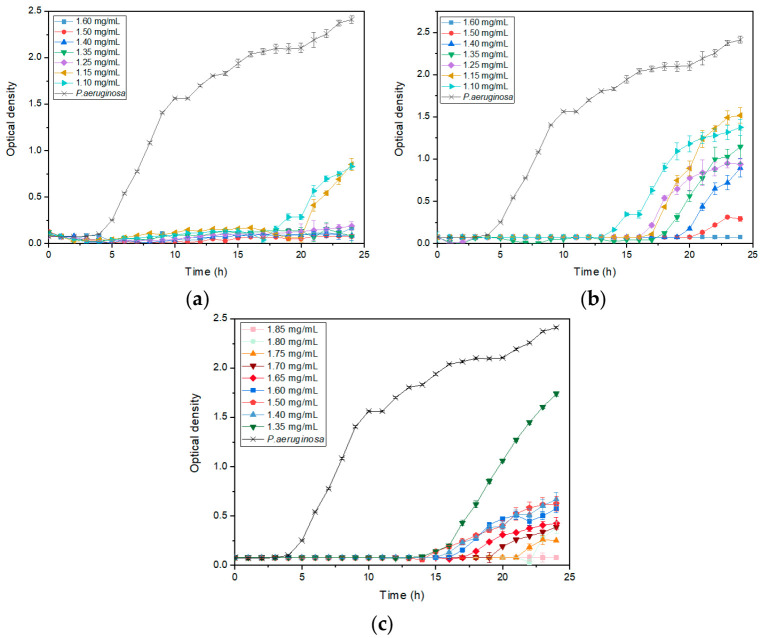
OD_600_ growth of *P. aeruginosa* in contact with (**a**) 22 nm; (**b**) 35 nm; and (**c**) 66 nm ZnO nanoparticles at different concentrations.

**Figure 5:**
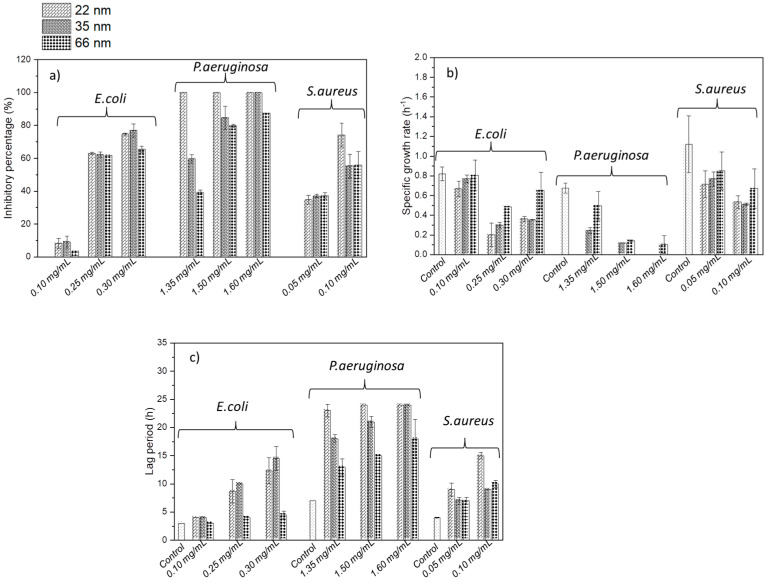
ZnO NP size effect on the growth parameter of *E. coli*; *P. aeruginosa*; and *S. aureus*: (**a**) inhibitory percentage; (**b**) specific growth rate; and (**c**) lag period at different concentrations.

Figure 4 shows the evolution of the optical density of P.aeruginosa bacteria in the presence of different NP sizes—22 nm (Figure 4a), 35 nm (Figure 4b), and 66 nm (Figure 4c)—at various concentrations. Increasing the concentration improved antibacterial activity, as the optical density was lower at higher concentrations. For NP concentrations where optical density did not remain constant over 24 h, the lag phase, during which the optical density remains at 0 before increasing, was longer with smaller NPs, indicating a stronger bacterial growth inhibition. For example, at a concentration of 1.35 mg/mL (in green in Figure 4), the lag phase of the bacteria with 22 nm NPs was longer than that with 35 nm, which was longer than that with 66 nm. Similarly, the growth rate, indicated by the slope, was higher with larger NPs, showing a faster bacterial growth and less inhibition. Additionally, the final bacterial density, related to the percentage inhibition, was higher with larger NPs. These observations enabled the calculation of these parameters and the evaluation of the impact of NP size on them. The effect of ZnO NP size on the lag phase, specific growth rate, and inhibition percentage for different bacteria is detailed in Figure 5.

The inhibition percentage generally increased with decreasing NP size, independently of bacteria (Figure 5a). While no significant impact is observed with *E. coli* and *S. aureus*, the influence of ZnO NP size is evident with *P. aeruginosa*. The 22 nm and 35 nm ZnO NPs showed similar inhibition levels on *E. coli* growth across tested concentrations except at 0.3 mg/mL, where inhibition percentages were 74.78%, 76.93%, and 65.59% for 22 nm, 35 nm, and 66 nm NPs, respectively.

The impact of NP size on inhibition percentage was particularly pronounced in P. aeruginosa across all concentrations tested. At 1.35 mg/mL, inhibition percentages decreased from 100%, 59.70% to 39.33% for 22 nm, 35 nm, and 66 nm NPs, respectively. Similarly, at 1.50 mg/mL, inhibition percentages decreased from 100%, 84.72% to 79.72% for the respective NPs sizes compared to the control. At 1.60 mg/mL, inhibition percentages decreased from 100% for 22 nm and 35 nm NPs to 87.59% for 66 nm NPs.

For *S. aureus*, inhibition percentages were identical for 35 nm and 66 nm NPs across all tested concentrations—37% for 35 nm and 66 nm NPs at 0.05 mg/mL, 34% for 22 nm NPs at 0.1 mg/mL, 56% at 0.1 mg/mL, and 74% for 22 nm NPs at 0.1 mg/mL.

Regarding the specific growth rate, representing bacterial proliferation rate, we observe a general trend across all microorganisms where smaller ZnO NPs decreased the specific growth rate. For *E. coli*, the specific growth rate was 0.67 h^−1^ with 22 nm NPs, compared to 0.8 h^−1^ with 35 nm and 66 nm NPs at 0.1 mg/mL concentration. At 0.25 mg/mL, it decreased to 0.2 h^−1^, 0.3 h^−1^, and 0.49 h^−1^ with 22 nm, 35 nm, and 66 nm NPs, respectively. At a 0.3 mg/mL NP concentration, the specific growth rate was 0.35 h^−1^ with 22 nm and 35 nm NPs, and 0.65 h^−1^ with 66 nm NPs.

*P. aeruginosa* exhibited rates of 0 h^−1^, 0.25 h^−1^, and 0.50 h^−1^ with 22 nm, 35 nm, and 66 nm NPs, respectively, at 1.35 mg/mL. At a 1.50 mg/mL concentration, the rates were 0 h^−1^, 0.12 h^−1^, and 0.14 h^−1^ with the respective NP sizes. Finally, at a concentration of 1.60 mg/mL, the specific growth rate of P. aeruginosa was 0 h^−1^ for 22 nm and 35 nm NPs, and 0.10 h^−1^ for 66 nm NPs.

For *S. aureus*, at 0.05 mg/mL, the specific growth rate was 0.71 h^−1^, 0.77 h^−1^, and 0.85 h^−1^ with 22 nm, 35 nm, and 66 nm NPs, respectively. On average, it was 0.52 h^−1^ with 22 nm and 35 nm NPs, and 0.67 h^−1^ with 66 nm NPs.

Further elucidation of the impact of NP size on bacterial growth kinetics was provided by examining the lag phase (Figure 5c). The lag phase, representing the time for bacteria to initiate growth after inoculation, also exhibits a general trend across all microorganisms, wherein smaller ZnO particles prolong the bacterial latency period. At 0.1 mg/mL, the lag phase remained stable across all sizes for *E. coli*, averaging 4 h. However, notable reductions were observed at higher concentrations. For instance, at 0.25 mg/mL, the lag phase of *E. coli* decreased from 8.5 h to 10 h to 4 h with 22 nm, 35 nm, and 66 nm NPs, respectively. Similarly, at 0.30 mg/mL, it decreased to 12 h, 14.5 h, and 4.5 h with the respective NPs sizes.

In the case of *P. aeruginosa*, at a concentration of 1.35 mg/mL, the lag phase reduced from 23 h to 13 h with 22 nm, 35 nm, and 66 nm NPs. This reduction was also observed at 1.50 mg/mL, where the lag phase was 24 h, 21 h, and 15 h with 22 nm, 35 nm, and 66 nm NPs, respectively. Similarly, at a 1.60 mg/mL NP concentration, the lag phase remained at 24 h for both 22 nm and 35 nm sizes, and decreased to 18 h for 66 nm NPs.

Similarly, for *S. aureus*, distinct differences in lag phase duration were observed at different concentrations and NPs sizes. At a 0.05 mg/mL concentration, the lag phase persisted for 9 h with 22 nm NPs, while it was reduced to 7 h with 35 nm and 66 nm NPs. Conversely, at a concentration of 0.1 mg/mL, the lag phase extended to 15 h with 22 nm NPs, while it remained at 10 h with 35 nm and 66 nm NPs.

**Figure 6:**
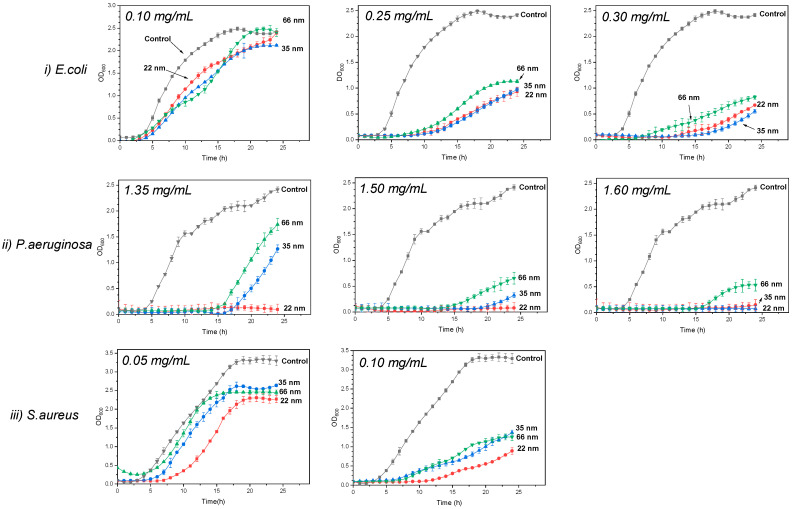
Effect of ZnO NP size on bacterial growth kinetics.

Figure 6 shows an increase in bacterial growth inhibition with decreasing NP size for all microorganisms at different ZnO NP concentrations. For *E. coli*, inhibition was consistent with 22 nm and 35 nm nanoparticles, and was higher than with 66 nm NPs. *P. aeruginosa* inhibition increases with decreasing size. Furthermore, as NP concentration increased, inhibition tended to converge for all three NP sizes. As for *S. aureus*, inhibition is consistent with 66 nm and 35 nm NPs, but weaker than contact with 22 nm NPs. These results confirm those obtained for growth parameters, MIC and MBC.

### 3.5. Antibacterial Activity of ZnO Nanoparticles on Emulsion Waterborne Paint

**Figure 7:**
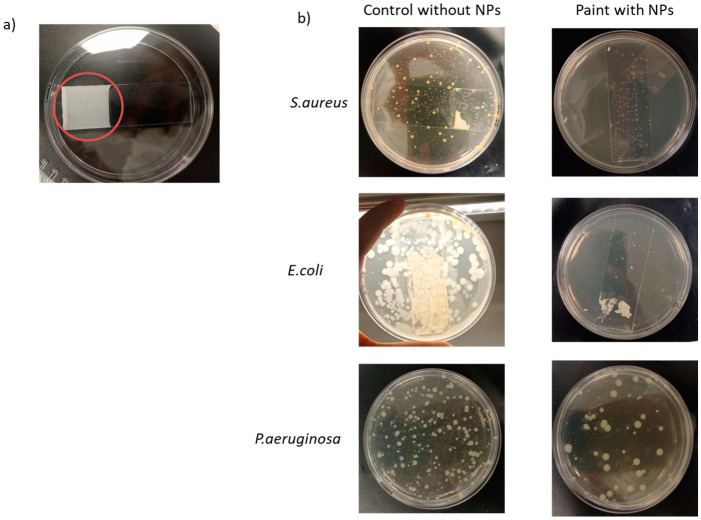
(**a**) A paint film containing 22 nm nanoparticles, onto which 0.1 mL of bacterial suspension is applied without vortexing; (**b**) evaluation of antibacterial properties of paint containing ZnO nanoparticles against *S. aureus*; *E. coli*; and *P. aeruginosa*.

The paint formulation containing 1wt% 22 nm ZnO NPs demonstrated a high efficacy against *S. aureus* and *E. coli*, while showing no antibacterial activity against *P. aeruginosa* (Figure 7). This trend was observed when NPs were in suspension, with *P. aeruginosa* exhibiting the lowest sensitivity to NPs. However, NPs in paint films exhibited a better antibacterial activity than in suspension. The number of colonies on agar plates seeded with samples diluted to 10^−4^ was compared to better observe the activity of NPs in paint on *P. aeruginosa*, unlike other bacteria where samples are directly presented on agar plates. The number of residual cells in Table 3 confirmed the inhibitory effect of NPs in the paint against *S. aureus* and *E. coli*, as well as the lack of inhibition against *P. aeruginosa*. Notably, paint’s detachment from slides occurred after the vortexing step used to remove bacteria from the glass slide.

## 4. Discussion

The most important findings of this study can be summarized as follows: Firstly, decreasing the size of ZnO NPs (22 nm, 35 nm, and 66 nm) enhanced their antibacterial efficacy by lowering their minimum inhibitory concentration (MIC) and minimum bactericidal concentration (MBC). Notably, *P. aeruginosa* exhibited a lower susceptibility to NPs than the other bacterial strains, necessitating higher concentrations for a bactericidal effect. Furthermore, the reduction in MIC and MBC with decreasing NPs size was particularly pronounced when dealing with *P. aeruginosa*.

Several investigations have explored the effects of ZnO NPs on MIC and MBC values [53,54,55,56,57,58]. Consistent with our findings, most researchers have observed a significant enhancement in antibacterial effectiveness with smaller NPs. For instance, Pasquet et al. [53] reported a superior antimicrobial activity with smaller ZnO crystals against *E. coli* and *P. aeruginosa*, noting a proportional increase in MIC and MBC with larger NPs.

Álvarez-Chimal et al. [59] have observed consistent findings, indicating that ZnO NPs sized at 7 nm, 10 nm, 16 nm, and 30 nm show increased MBC values against both *S. aureus* and *E. coli* as their size increases. Remarkably, the MBC values reported in their study, ranging from 10 to 80 mg/mL, are higher than those observed in the present investigation, likely due to differences in NP composition. However, our results regarding *S. aureus* diverge from those of Lallo Da Silva et al. [38], who have documented elevated MIC values with larger NPs against *S. aureus* strains. Palanikumar et al. [55] reported that ZnO NPs with sizes of 15 nm, 25 nm, and 38 nm exhibited identical MICs against *S. aureus* MRSA, with a value of 0.2 mg/mL, aligning with our findings. Despite *S. aureus* consistently displaying similar MIC values regardless of NP size, we observed a decline in cell viability, as indicated by MBC measurements, with decreasing particle size. The MIC and MBC values obtained in this study are less than or equal to those reported in the literature for ZnO NPs of the same size as those studied here [32,60].

Previous studies have suggested that *E. coli* is more susceptible to ZnO NPs than *P. aeruginosa*, while *S. aureus* is more sensitive than *P. aeruginosa*. The variation in susceptibility among *P. aeruginosa* with other bacteria can be attributed to a complex interplay of factors, including the production of extracellular polymeric substances (EPSs) [61], detoxification systems, and specific metabolic and genetic responses. The extensive coding capacity of the *P. aeruginosa* genome enables remarkable metabolic adaptability and versatility in response to environmental changes [62]. Factors dictating the antibacterial effect can explain the difference in sensitivity of *S. aureus* and *E.coli* towards ZnO, specifically the structural differences between Gram(-) and Gram(+) bacteria. In Gram(+) bacteria, such as *S. aureus*, there is no outer membrane, and the cell wall is thick, consisting of a large amount of mucopeptides as well as surface components of lipoteichoic acids (LTAs). In contrast, Gram(-) bacteria, like *E.coli*, have a relatively thin cell wall but possess an outer membrane. The lack of an outer membrane could explain why *S. aureus* were more sensitive to ZnO NPs than *E. coli*.

Secondly, the study suggested that the diminution in the size of ZnO NPs can slow down bacterial growth. Few studies have delved into the role of ZnO NP size in the kinetic growth of bacteria. Nevertheless, Baek et al. discovered that the growth inhibition rate (%) of *E. coli* increased as the size of ZnO NPs decreased (12.7 nm, 15.7 nm, and 17.2 nm) [63]. These results align with those of Mirhosseini et al. [48] and Raghupathi et al. [49], who have similarly observed increased growth inhibition against *S. aureus* and *E. coli* with smaller ZnO NP sizes.

The antibacterial activity of ZnO NPs is attributed to several mechanisms, including releasing reactive oxygen species (ROS), destroying the cell membrane, and internalizing NPs into bacterial cells. The reduction in NP size can influence these mechanisms and slow bacterial growth in several ways. The heightened reactivity of smaller-sized NPs due to their larger specific surface area can promote increased ROS release upon interaction with bacteria, leading to the greater oxidation and deterioration of essential cellular components, thus slowing bacterial growth. Also, the reduced size of NPs can result in more efficient internalization into bacterial cells. Lastly, the decrease in NP size can also increase the likelihood of direct contact with the cell membrane, potentially causing more significant membrane disruption and the leakage of cellular components, thereby compromising bacterial viability. Furthermore, the similar MIC values of NPs against *S. aureus* may be attributed to the fact that *S. aureus* is a cocci-shaped bacteria that tends to form “grape-like” clusters. The internal cocci in these clusters could explain these observations. There appears to be a maximum size beyond which NPs cannot penetrate between these grape-like clusters, thus maintaining unchanged antibacterial activity. Furthermore, the inhibitory effects of NPs on *P. aeruginosa* growth tend to converge as their concentration increases, probably because the minimum inhibitory concentration (MIC) is close.

Thirdly, ZnO nano-based paints exhibit bactericidal activity against *E. coli* and *S. aureus*. These findings align with the literature, demonstrating the effectiveness of paints containing ZnO nanoparticles against various bacteria, including *E. coli* and *S. aureus* [64,65,66]. For example, Fiori et al. [64] found that the most significant antimicrobial effect against *S. aureus*, assessed using an agar diffusion test, was achieved with paint formulated with 1.2% 9 nm ZnO NPs. The antibacterial activity of NPs was higher when incorporated in paint, probably due to the optimization of the NP–cell interaction surface.

## 5. Conclusions

The reduction in the size of ZnO nanoparticles enhanced their antibacterial activity against *E. coli*, *P. aeruginosa*, and *S. aureus*. Additionally, it decreased the growth kinetics of bacteria by reducing their specific growth rate, prolonging their lag time, and increasing their inhibition percentage. For example, in *P. aeruginosa*, nanoparticle concentrations at 1.35 mg/mL showed inhibition percentages that decreased from 100% for 22 nm NPs, to 59.70% for 35 nm NPs and 39.33% for 66 nm NPs. This study emphasizes the critical importance of ZnO NP size in their ability to inhibit and eradicate pathogenic Gram-negative and Gram-positive bacteria. Furthermore, applying these ZnO nanoparticles in paint formulations presented a promising strategy to combat nosocomial infections. A prospective direction for this study is to investigate the antibacterial activity of their thin films and the safety of ZnO NPs.

## Figures and Tables

**Table 1:** Synthesis parameters of ZnO nanopowders.

Sample Size	[Zn^2+^]/[MEA]	[Zn^2+^]/[H_2_O]	Agitation Time	Temperature
22 nm	1	5	22 h	80 °C
35 nm	1	10	2 h	110 °C
66 nm	2	10	22 h	110 °C

**Table 2:** Crystallographic parameters of the obtained ZnO nanoparticles.

Crystallite Size (nm)	Lattice Parameters (Å)		Microstrain (%)	Sig = GoF
	a = b	c		
22	3.25	5.20	0.002	1.18
35	3.25	5.21	0.001	1.21
66	3.25	5.21	0.001	1.60

**Table 3:** Residual survival of bacteria after 24 h of contact with paint.

Bacteria	Control at 24 h	Sample at 24 h
***S. aureus***	4.55 ± 0.32 Log CFU/cm^2^	<1 Log_10_ CFU/cm^2^
***E. coli***	3.90 ± 0.02 Log_10_ CFU/cm^2^	<1 Log_10_ CFU/cm^2^
***P. aeruginosa***	6.41 ± 0.11 Log_10_ CFU/cm^2^	6.18 ± 0.08 Log_10_ CFU/cm^2^

## Data Availability

The corresponding author will make the raw data supporting this article’s conclusions available upon request.

## References

[1] Baviskar A.S., Khatib K.I., Rajpal D., Dongare H.C. (2019). Nosocomial Infections in Surgical Intensive Care Unit: A Retrospective Single-Center Study. Int. J. Crit. Illn. Inj. Sci..

[2] Dadgostar P. (2019). Antimicrobial Resistance: Implications and Costs. Infect. Drug Resist..

[3] Ajulo S., Awosile B. (2024). Global Antimicrobial Resistance and Use Surveillance System (GLASS 2022): Investigating the Relationship between Antimicrobial Resistance and Antimicrobial Consumption Data across the Participating Countries. PLoS ONE.

[4] Morrison L., Zembower T.R. (2020). Antimicrobial Resistance. Gastrointest. Endosc. Clin. N. Am..

[5] Strengthening the EU Response to Prevention and Control of Antimicrobial Resistance (AMR): Policy Priorities for Effective Implementation Strengthening the EU Response to Prevention and Control of Antimicrobial Resistance (AMR): Policy Priorities for Effective Implementation. https://eurohealthobservatory.who.int/publications/i/strengthening-the-eu-response-to-prevention-and-control-of-antimicrobial-resistance-(amr)-policy-priorities-for-effective-implementation.

[6] Raghunath A., Perumal E. (2017). Metal Oxide Nanoparticles as Antimicrobial Agents: A Promise for the Future. Int. J. Antimicrob. Agents.

[7] Pachaiappan R., Rajendran S., Show P.L., Manavalan K., Naushad M. (2021). Metal/Metal Oxide Nanocomposites for Bactericidal Effect: A Review. Chemosphere.

[8] Tiwari V., Mishra N., Gadani K., Solanki P.S., Shah N.A., Tiwari M. (2018). Mechanism of Anti-Bacterial Activity of Zinc Oxide Nanoparticle Against Carbapenem-Resistant *Acinetobacter baumannii*. Front. Microbiol..

[9] Gautam S., Das D.K., Kaur J., Kumar A., Ubaidullah M., Hasan M., Yadav K.K., Gupta R.K. (2023). Transition Metal-Based Nanoparticles as Potential Antimicrobial Agents: Recent Advancements, Mechanistic, Challenges, and Future Prospects. Discov. Nano.

[10] Sukhanova A., Bozrova S., Sokolov P., Berestovoy M., Karaulov A., Nabiev I. (2018). Dependence of Nanoparticle Toxicity on Their Physical and Chemical Properties. Nanoscale Res. Lett..

[11] Raha S., Ahmaruzzaman M. (2022). ZnO Nanostructured Materials and Their Potential Applications: Progress, Challenges and Perspectives. Nanoscale Adv..

[12] Sarojini S., Jayaram S., Pal K. (2021). An Impact of Antibacterial Efficacy of Metal Oxide Nanoparticles: A Promise for Future. Bio-Manufactured Nanomaterials: Perspectives and Promotion.

[13] Jiang S., Lin K., Cai M. (2020). ZnO Nanomaterials: Current Advancements in Antibacterial Mechanisms and Applications. Front. Chem..

[14] Izzi M., Sportelli M.C., Torsi L., Picca R.A., Cioffi N. (2023). Synthesis and Antimicrobial Applications of ZnO Nanostructures: A Review. ACS Appl. Nano Mater..

[15] Dutta G., Sugumaran A. (2021). Bioengineered Zinc Oxide Nanoparticles: Chemical, Green, Biological Fabrication Methods and Its Potential Biomedical Applications. J. Drug Deliv. Sci. Technol..

[16] Mendes C.R., Dilarri G., Forsan C.F., Sapata V.d.M.R., Lopes P.R.M., de Moraes P.B., Montagnolli R.N., Ferreira H., Bidoia E.D. (2022). Antibacterial Action and Target Mechanisms of Zinc Oxide Nanoparticles against Bacterial Pathogens. Sci. Rep..

[17] Ahmad I., Alshahrani M.Y., Wahab S., Al-Harbi A.I., Nisar N., Alraey Y., Alqahtani A., Mir M.A., Irfan S., Saeed M. (2022). Zinc Oxide Nanoparticle: An Effective Antibacterial Agent against Pathogenic Bacterial Isolates. J. King Saud. Univ. Sci..

[18] Perveen R., Shujaat S., Qureshi Z., Nawaz S., Khan M.I., Iqbal M. (2020). Green versus Sol-Gel Synthesis of ZnO Nanoparticles and Antimicrobial Activity Evaluation against Panel of Pathogens. J. Mater. Res. Technol..

[19] Abdal Dayem A., Hossain M.K., Lee S.B., Kim K., Saha S.K., Yang G.-M., Choi H.Y., Cho S.-G. (2017). The Role of Reactive Oxygen Species (ROS) in the Biological Activities of Metallic Nanoparticles. Int. J. Mol. Sci..

[20] Babayevska N., Przysiecka Ł., Iatsunskyi I., Nowaczyk G., Jarek M., Janiszewska E., Jurga S. (2022). ZnO Size and Shape Effect on Antibacterial Activity and Cytotoxicity Profile. Sci. Rep..

[21] Girma A., Abera B., Mekuye B., Mebratie G. (2024). Antibacterial Activity and Mechanisms of Action of Inorganic Nanoparticles against Foodborne Bacterial Pathogens: A Systematic Review. IET Nanobiotechnol..

[22] Bhattacharya P., Dey A., Neogi S. (2021). An Insight into the Mechanism of Antibacterial Activity by Magnesium Oxide Nanoparticles. J. Mater. Chem. B.

[23] Kessler A., Hedberg J., Blomberg E., Odnevall I. (2022). Reactive Oxygen Species Formed by Metal and Metal Oxide Nanoparticles in Physiological Media—A Review of Reactions of Importance to Nanotoxicity and Proposal for Categorization. Nanomaterials.

[24] Abebe B., Zereffa E.A., Tadesse A., Murthy H.C.A. (2020). A Review on Enhancing the Antibacterial Activity of ZnO: Mechanisms and Microscopic Investigation. Nanoscale Res. Lett..

[25] Agarwal H., Menon S., Venkat Kumar S., Rajeshkumar S. (2018). Mechanistic Study on Antibacterial Action of Zinc Oxide Nanoparticles Synthesized Using Green Route. Chem. Biol. Interact..

[26] Modi S.K., Gaur S., Sengupta M., Singh M.S. (2023). Mechanistic Insights into Nanoparticle Surface-Bacterial Membrane Interactions in Overcoming Antibiotic Resistance. Front. Microbiol..

[27] Raghav A., Kaur S., Setia G., Kumar S., Shah M.P., Bharadvaja N., Kumar L. (2024). Nanomaterials Induced Cell Disruption: An Insight into Mechanism. Biogenic Nanomaterials for Environmental Sustainability: Principles, Practices, and Opportunities.

[28] Thakur S., Neogi S. (2021). Effect of Doped ZnO Nanoparticles on Bacterial Cell Morphology and Biochemical Composition. Appl. Nanosci..

[29] Xin Z., He Q., Wang S., Han X., Fu Z., Xu X., Zhao X. (2022). Recent Progress in ZnO-Based Nanostructures for Photocatalytic Antimicrobial in Water Treatment: A Review. Appl. Sci..

[30] Kaur H., Rauwel P., Rauwel E., Guisbiers G. (2023). Chapter 6—Antimicrobial Nanoparticles: Synthesis, Mechanism of Actions. Antimicrobial Activity of Nanoparticles.

[31] Ghaffari S.-B., Sarrafzadeh M.-H., Salami M., Alvandi A. (2024). A Comparative Study of the Action Mechanisms and Development Strategies of Different ZnO-Based Nanostructures in Antibacterial and Anticancer Applications. J. Drug Deliv. Sci. Technol..

[32] Sharma S., Kumar K., Thakur N., Chauhan S., Chauhan M.S. (2020). The Effect of Shape and Size of ZnO Nanoparticles on Their Antimicrobial and Photocatalytic Activities: A Green Approach. Bull. Mater. Sci..

[33] Jaiswal P.B., Jejurikar S., Mondal A., Pushkar B., Mazumdar S. (2023). Antibacterial Effects of ZnO Nanodisks: Shape Effect of the Nanostructure on the Lethality in *Escherichia coli*. Appl. Biochem. Biotechnol..

[34] Motelica L., Oprea O.-C., Vasile B.-S., Ficai A., Ficai D., Andronescu E., Holban A.M. (2023). Antibacterial Activity of Solvothermal Obtained ZnO Nanoparticles with Different Morphology and Photocatalytic Activity against a Dye Mixture: Methylene Blue, Rhodamine B and Methyl Orange. Int. J. Mol. Sci..

[35] Zubair N., Akhtar K. (2020). Morphology Controlled Synthesis of ZnO Nanoparticles for In-Vitro Evaluation of Antibacterial Activity. Trans. Nonferrous Met. Soc. China.

[36] Gharpure S., Ankamwar B. (2020). Synthesis and Antimicrobial Properties of Zinc Oxide Nanoparticles. J. Nanosci. Nanotechnol..

[37] Droepenu E.K., Amenyogbe E., Boatemaa M.A., Opoku E. (2024). Study of the Antimicrobial Activity of Zinc Oxide Nanostructures Mediated by Two Morphological Structures of Leaf Extracts of *Eucalyptus robusta* Sm. Heliyon.

[38] Lallo da Silva B., Caetano B.L., Chiari-Andréo B.G., Pietro R.C.L.R., Chiavacci L.A. (2019). Increased Antibacterial Activity of ZnO Nanoparticles: Influence of Size and Surface Modification. Colloids Surf. B Biointerfaces.

[39] Bouttier-Figueroa D.C., Cortez-Valadez M., Flores-Acosta M., Robles-Zepeda R.E. (2024). Green Synthesis of Zinc Oxide Nanoparticles Using Plant Extracts and Their Antimicrobial Activity. BioNanoSci.

[40] Aldeen T.S., Ahmed Mohamed H.E., Maaza M. (2022). ZnO Nanoparticles Prepared via a Green Synthesis Approach: Physical Properties, Photocatalytic and Antibacterial Activity. J. Phys. Chem. Solids.

[41] Awasthi A., Sharma P., Jangir L., Kamakshi, Awasthi G., Awasthi K.K., Awasthi K. (2020). Dose Dependent Enhanced Antibacterial Effects and Reduced Biofilm Activity against *Bacillus Subtilis* in Presence of ZnO Nanoparticles. Mater. Sci. Eng. C.

[42] Dadi R., Kerignard E., Traoré M., Mielcareck C., Kanaev A., Azouani R. (2021). Evaluation of Antibacterial Efficiency of Zinc Oxide Thin Films Nanoparticles against Nosocomial Bacterial Strains. Chem. Eng. Trans..

[43] Wang F., Qi J., Zhu L. (2024). Ag/MoS_2_ Nanozyme-Modified ZnO Nanopillar Surface for Enhanced Synergistic Mechanical and Chemical Antibacterial Activity. Colloids Surf. A Physicochem. Eng. Asp..

[44] Zaman Y., Ishaque M.Z., Waris K., Shahzad M., Siddique A.B., Arshad M.I., Zaman H., Ali H.M., Kanwal F., Aslam M. (2023). Modified Physical Properties of Ni Doped ZnO NPs as Potential Photocatalyst and Antibacterial Agents. Arab. J. Chem..

[45] Xiang E., Moran C.S., Ivanovski S., Abdal-hay A. (2023). Nanosurface Texturing for Enhancing the Antibacterial Effect of Biodegradable Metal Zinc: Surface Modifications. Nanomaterials.

[46] Xu L., Liang H.-W., Yang Y., Yu S.-H. (2018). Stability and Reactivity: Positive and Negative Aspects for Nanoparticle Processing. Chem. Rev..

[47] Amodeo J., Pizzagalli L. (2021). Modeling the Mechanical Properties of Nanoparticles: A Review. C. R. Phys..

[48] Mirhosseini F., Amiri M., Daneshkazemi A., Zandi H., Javadi Z.S. (2019). Antimicrobial Effect of Different Sizes of Nano Zinc Oxide on Oral Microorganisms. Front. Dent..

[49] Raghupathi K.R., Koodali R.T., Manna A.C. (2011). Size-Dependent Bacterial Growth Inhibition and Mechanism of Antibacterial Activity of Zinc Oxide Nanoparticles. Langmuir.

[50] El-Habib I., Roynette A., Morakchi-Goudjil H., Lemarchand A., Christine M., Azouani R., Traore M. (2023). Synthesis by Soft Chemistry of Size-Controlled Zinc Oxide (ZnO) Nanocrystals for Antimicrobial Applications. MATEC Web Conf..

[51] Luo Z., Zhu M., Guo M., Lian Z., Tong W., Wang J., Zhang B., Wei W. (2018). Ultrasonic-Assisted Dispersion of ZnO Nanoparticles and Its Inhibition Activity to *Trichoderma viride*. J. Nanosci. Nanotechnol..

[52] (2011). Measurement of Antibacterial Activity on Plastics and Other Non-Porous Surfaces.

[53] Pasquet J., Chevalier Y., Couval E., Bouvier D., Noizet G., Morlière C., Bolzinger M.-A. (2014). Antimicrobial Activity of Zinc Oxide Particles on Five Micro-Organisms of the Challenge Tests Related to Their Physicochemical Properties. Int. J. Pharm..

[54] Jones N., Ray B., Ranjit K.T., Manna A.C. (2008). Antibacterial Activity of ZnO Nanoparticle Suspensions on a Broad Spectrum of Microorganisms. FEMS Microbiol. Lett..

[55] Palanikumar L., Ramasamy S.N., Balachandran C. (2014). Size-Dependent Antimicrobial Response of Zinc Oxide Nanoparticles. IET Nanobiotechnol..

[56] Azam A., Ahmed A.S., Oves M., Khan M., Memic A. (2012). Size-Dependent Antimicrobial Properties of CuO Nanoparticles against Gram-Positive and -Negative Bacterial Strains. Int. J. Nanomed..

[57] Lallo da Silva B., Abuçafy M.P., Berbel Manaia E., Oshiro Junior J.A., Chiari-Andréo B.G., Pietro R.C.R., Chiavacci L.A. (2019). Relationship Between Structure And Antimicrobial Activity Of Zinc Oxide Nanoparticles: An Overview. Int. J. Nanomed..

[58] Applerot G., Lipovsky A., Dror R., Perkas N., Nitzan Y., Lubart R., Gedanken A. (2009). Enhanced Antibacterial Activity of Nanocrystalline ZnO Due to Increased ROS-Mediated Cell Injury. Adv. Funct. Mater..

[59] Álvarez-Chimal R., García-Pérez V.I., Álvarez-Pérez M.A., Tavera-Hernández R., Reyes-Carmona L., Martínez-Hernández M., Arenas-Alatorre J.Á. (2022). Influence of the Particle Size on the Antibacterial Activity of Green Synthesized Zinc Oxide Nanoparticles Using *Dysphania Ambrosioides* Extract, Supported by Molecular Docking Analysis. Arab. J. Chem..

[60] Aldin K.S., Al-Hariri S., Ali-Nizam A. (2020). Effectiveness of ZnO Nano Particles against the Foodborne Microbial Pathogens *E. coli* and *S. aureus*. Jordan J. Chem. (JJC).

[61] Grossich R., Lemos Vilches M., Costa C.S., Pezzoni M. (2023). Role of Pel and Psl Polysaccharides in the Response of *Pseudomonas aeruginosa* to Environmental Challenges: Oxidative Stress Agents (UVA, H_2_O_2_, Sodium Hypochlorite) and Its Competitor *Staphylococcus aureus*. Microbiology.

[62] Pang Z., Raudonis R., Glick B.R., Lin T.-J., Cheng Z. (2019). Antibiotic Resistance in Pseudomonas Aeruginosa: Mechanisms and Alternative Therapeutic Strategies. Biotechnol. Adv..

[63] Baek S., Joo S.H., Kumar N., Toborek M. (2017). Antibacterial Effect and Toxicity Pathways of Industrial and Sunscreen ZnO Nanoparticles on *Escherichia coli*. J. Environ. Chem. Eng..

[64] Fiori J., Silva L., Picolli K.C., Ternus R., Ilha J., Decalton F., de Mello J.M., Riella H., Fiori M. (2017). Zinc Oxide Nanoparticles as Antimicrobial Additive for Acrylic Paint. Mater. Sci. Forum.

[65] Agustin W., Albari M.T., Ghifari M.A., Ghifari M.R., Purnamasari D., Mandeli R.S. (2024). The Antibacterial Properties of Paint with the Addition of ZnO Nanoparticles. AIP Conf. Proc..

[66] Foudi H., Soukeur A., Rekhila G., Trari M., Amara M. (2023). Synthesis and Characterization of ZnO Nanoparticles for Antibacterial Paints. Chem. Pap..

